# Addressing the inconsistent electric fields of tDCS by using patient-tailored configurations in chronic stroke: Implications for treatment

**DOI:** 10.1016/j.nicl.2022.103178

**Published:** 2022-08-29

**Authors:** Joris van der Cruijsen, Renée F. Dooren, Alfred C. Schouten, Thom F. Oostendorp, Maarten A. Frens, Gerard M. Ribbers, Frans C.T. van der Helm, Gert Kwakkel, Ruud W. Selles, on behalf of the 4D EEG consortium

**Affiliations:** a Erasmus MC, University Medical Center Rotterdam, dept. of Rehabilitation Medicine, Doctor Molewaterplein 40, 3015 GD, Rotterdam, The Netherlands; b Delft University of Technology, dept. of Biomechanical Engineering, Mekelweg 2, 2628 CD, Delft, The Netherlands; c Radboud University Medical Center, dept. of Rehabilitation, Reinier Postlaan 2, 6525 GC, Nijmegen, The Netherlands; d University of Twente, dept. of Biomechanical Engineering, Drienerlolaan 5, 7522 NB, Enschede, The Netherlands; e Donders Institute for Brain, Cognition and Behaviour, Kapittelweg 29, 6525 EN, Nijmegen, The Netherlands; f Rijndam Rehabilitation, Westersingel 300, 3015 LJ, Rotterdam, The Netherlands; g Northwestern University of Chicago, dept. of Physical Therapy and Movement Sciences, 420 E Superior St, Chicago, IL 60611, United States; h Amsterdam University Medical Centre, dept. of Rehabilitation Medicine, De Boelelaan 1117, 1118, 1081 HV Amsterdam, The Netherlands

**Keywords:** Stroke, tDCS, Volume conduction models, EEG, Source localization

## Abstract

Transcranial direct current stimulation (tDCS) is a promising tool to improve and speed up motor rehabilitation after stroke, but inconsistent clinical effects refrain tDCS from clinical implementation. Therefore, this study aimed to assess the need for individualized tDCS configurations in stroke, considering interindividual variability in brain anatomy and motor function representation.

We simulated tDCS in individualized MRI-based finite element head models of 21 chronic stroke subjects and 10 healthy age-matched controls. An anatomy-based stimulation target, i.e. the motor hand knob, was identified with MRI, whereas a motor function-based stimulation target was identified with EEG. For each subject, we simulated conventional anodal tDCS electrode configurations and optimized electrode configurations to maximize stimulation strength within the anatomical and functional target. The normal component of the electric field was extracted and compared between subjects with stroke and healthy, age-matched controls, for both targets, during conventional and optimized tDCS.

Electrical field strength was significantly lower, more variable and more frequently in opposite polarity for subjects with stroke compared to healthy age-matched subjects, both for the anatomical and functional target with conventional, i.e. non-individualized, electrode configurations. Optimized, i.e. individualized, electrode configurations increased the electrical field strength in the anatomical and functional target for subjects with stroke but did not reach the same levels as in healthy subjects.

Considering individual brain structure and motor function is crucial for applying tDCS in subjects with stroke. Lack of individualized tDCS configurations in subjects with stroke results in lower electric fields in stimulation targets, which may partially explain the inconsistent clinical effects of tDCS in stroke trials.

## Highlights

• Conventional anodal M1 tDCS leads to variable electric fields in stroke patients.
• Optimizing electrode locations increases stimulation strength in stroke patients.
• Individual brain anatomy and function require consideration in tDCS for stroke patients.
• Lack of individualization may partially explain mixed tDCS effects in clinical RCTs.

## 1 Introduction

Transcranial direct current stimulation (tDCS) is a promising tool to speed up and improve motor rehabilitation after stroke (Hamoudi et al., 2018, Schlaug et al., 2008) but inconsistent effects refrain tDCS from clinical implementation. (Bornheim et al., 2020, Chhatbar et al., 2016, Elsner et al., 2017, Hashemirad et al., 2016, Lefebvre and Liew, 2017, Santos Ferreira et al., 2019) The rationale behind tDCS in post stroke motor rehabilitation is to drive an electric current through regions involved in a specific motor task, such as the primary motor cortex (M1) or premotor cortex (Hamoudi et al., 2018, Schlaug et al., 2008). TDCS is suggested to increase synaptic plasticity and consequently boost motor learning (Stagg and Nitsche, 2011). However, several *meta*-analyses show inconsistent effects of tDCS on motor recovery after stroke, with a wide range of effect sizes between studies (Bornheim et al., 2020, Chhatbar et al., 2016, Elsner et al., 2017, Hashemirad et al., 2016, Lefebvre and Liew, 2017, Santos Ferreira et al., 2019).

Inconsistent tDCS effects in stroke randomized controlled trials (RCTs) may be explained by structural variability due to stroke lesions, resulting in alterations in local conductivity. Variability in conductivity can lead to differences in the electric current pathways in the brain between healthy subjects and subjects with stroke and within subjects with stroke, depending on the lesion’s location, size, and conductivity (Dmochowski et al., 2013, Johnstone et al., 2021, Minjoli et al., 2017, Piastra et al., 2021).

Another possible factor contributing to inconsistent tDCS effects in subjects with stroke is that the stroke lesion causes functional reorganization (Jones and Adkins, 2015, Volz et al., 2015, Ward, 2005) which may change the brain areas that tDCS should target. Functional reorganization of motor areas following stroke may involve the ipsilesional dorsal premotor cortex (Fridman et al., 2004, O’Shea et al., 2007, Ward, 2011, Werhahn et al., 2003) and the contralesional primary motor cortex (Murase et al., 2004, Volz et al., 2015); areas not targeted with conventional tDCS electrode configurations.

Currently, it is unknown to what extent conventional tDCS protocols in subjects with stroke are robust to structural and functional interindividual variability or whether the tDCS electrode configurations need to be individualized. Yet, previous simulation studies showed that conventional tDCS resulted in highly variable electric fields within the motor hand knob in patients with chronic (Minjoli et al., 2017, Piastra et al., 2021). Clinical studies have used conventional electrode positions to stimulate the ipsilesional M1 and/or simultaneously suppressing the contralesional M1 or by stimulating the ipsilesional premotor cortex and found improved motor skill acquisition and gains in clinical outcome measures of variable effect sizes (Andrade et al., 2017, Bolognini et al., 2011, Elsner et al., 2017, Lefebvre et al., 2013, Lindenberg et al., 2010). None of these studies addressed the structural *and* functional variability within subjects with stroke. Thus, it remains unclear if the combined effect of structural and functional interindividual variability could explain the variable tDCS effects sizes in stroke subjects.

The goal of our study was to assess the need for individualizing tDCS configurations in subjects with stroke by: 1) evaluating the electric field strength of conventional tDCS electrode configurations in subjects with stroke and healthy age-matched controls, taking into account individual brain structure and functional organization, and 2) identifying optimal individual tDCS electrode configurations based on individual brain structure and functional organization, and 3) evaluating the electric field strength of these optimized configurations in the anatomical and functional targets. To do so, we first simulated the electric fields generated by conventional anodal tDCS protocols in a target based on structural imaging (anatomical target) and in a target based on functional neuroimaging (functional target) recorded during a motor task. Second, we identified the optimal individual tDCS electrode configuration corresponding to the maximal achievable electric field strength in both targets and subsequently, compared the field strength obtained with these configurations in each stroke patient and healthy age-matched subjects.

## 2 Methods

The data in this study were collected by the 4D EEG consortium (Vlaar et al., 2017). A full description of the participants and the experimental design for collecting the EEG and MRI data was described in the study of Vlaar et al. (2017) (Vlaar et al., 2017) and will be summarized below. The Medical Ethics Reviewing Committee of the VU University Medical Centre (Amsterdam, the Netherlands) approved the study (NL47079.029.14). All experimental procedures complied with the Declaration of Helsinki.

### 2.1 Recruitment and clinical assessments

This study includes 21 chronic stroke patients (i.e., at least 6 months post-stroke at the time of inclusion, with initial hemiparesis). The full recruitment procedure is described by Vlaar et al. (2017) (Vlaar et al., 2017). Upper extremity motor function was assessed for the stroke patients at the time of inclusion using the Fugl-Meyer Assessment of the upper extremity (FM-UE) (Fugl-Meyer et al., 1975) and sensory function with the Erasmus modified Nottingham Sensory Assessment (EmNSA) (Stolk-Hornsveld et al., 2006). As a control group, 10 healthy, age-matched subjects were recruited.

### 2.2 Head models

To create individualized head models for tDCS simulation and EEG source localization, we used structural T1w MRIs of each participant. All T1w MRIs were acquired at the VU University Medical Center, Amsterdam, using a Discovery MR750 3 T scanner (GE, Waukesha, WI, USA) with a 3D fast spoiled gradient-recalled-echo sequence, consisting of 172 sagittal slices (256 × 256), using the following acquisition parameters: TR = 8.208 ms, TE = 3.22 ms, inversion time = 450 ms, flip angle = 12°, voxel size 1 × 0.94 × 0.94 mm (Vlaar et al., 2017). Locations of the nasion and the preauricular points were visually identified from the MRI to align the EEG cap.

SimNIBS 3.2 was used to create finite element volume conductor models of the head for simulation of non-invasive brain stimulation (Thielscher et al., 2015). The head models generated by SimNIBS were created using *headreco* (Nielsen et al., 2018) and *CAT12,* and consisted of six different tissue types: eyes, skin, skull, cerebrospinal fluid (CSF), grey matter and white matter. Tetrahedral meshes were created with default settings, resulting in 3.93 million (range: [3.46 to 4.62 million]) tetrahedral elements on average. Computational time was 2:45 h for *headreco* and 20 min for calculating the leadfield (using the *Pardiso* solver) required for optimization on a mobile computer (Windows 11, AMD Ryzen 9 5900HX, 32 GB RAM).

We incorporated the stroke lesions in the model by applying the LINDA algorithm (Pustina et al., 2016) on the T1w MRI to define a lesion mask. Then, we relabelled any CSF/grey matter/white matter elements of the SimNIBS model that overlapped with the lesion mask to ‘lesion’, resulting in a 7-tissue head model (Piastra et al., 2021). Conductivity values of all tissues were set at: eyes: 0.500 S/m; skin: 0.465 S/m; skull 0.010 S/m; CSF: 1.654 S/m; grey matter: 0.275 S/m; white matter: 0.126 S/m) and set the lesion conductivity equal to CSF conductivity (1.654 S/m).

### 2.3 Stimulation targets

To quantify the stimulation strength of all simulated tDCS configurations, we evaluated the normal component of the simulated electric field in the middle layer of the grey matter. This normal component was calculated for different tDCS configurations in stimulation targets based on either brain anatomy or functional motor organization.

#### 2.3.1 Anatomical target

To identify the individual anatomical tDCS targets, we visually identified the motor hand knob (from now on referred to as the anatomical target) on the T1w MRI for all subjects. The hand knob has an interindividual consistent folding pattern, with a small variety of typical hand knob structures (Caulo et al., 2007). As such, it can be determined from the T1w MRI (Caulo et al., 2007). We extracted the coordinates of the anterior side of the hand knob as the anatomical target. The anterior side was preferred above the posterior side due to its more prominent role in movement initiation (Terumitsu et al., 2009).

#### 2.3.2 Functional target

To identify the individual functional motor tDCS targets, we analyzed EEG recorded with 62 Ag/AgCl electrodes (TMSi, the Netherlands) while participants performed a robotic wrist-manipulator task. The EEG cap was arranged according to the international 10/10 system (Oostenveld and Praamstra, 2001) and recorded by a biosignal amplifier (Refa128, TMSi). All data were recorded at 2048 Hz, with only an anti-aliasing filter. A snap-on electrode at the left mastoid served as the ground electrode. The impedance of all EEG electrodes was below 20 kOhm before the experiment started. In addition, all electrode positions, the nasion, and both preauricular points were digitized for co-registration with the MRI.

To evoke cortical activity, the robotic wrist-manipulator continuously perturbed the impaired wrist of the stroke patients and the dominant right hand of the healthy controls during a passive and an active task. In the passive task, participants relaxed their wrist, following the motion of the manipulator to stimulate the somatosensory system. During the active task, participants maintained a wrist flexion torque of 20 % of the maximum voluntary contraction to elicit motor activity. The maximum voluntary contraction was determined per subject for the perturbed arm.

Participants performed 20 trials of 12.5 s for each task. Each single trial consisted of 10 repetitions of 1.25 s of the same perturbation. One stroke patient was not able to perform the active motor task. After cleaning the data, an average of 136 (range: 84 to 184) and 124 (range: 0 to 206) repetitions remained for the stroke patients' passive and active tasks, respectively. An average of 133 (range: 99 to 182) and 144 (range: 96 to 177) repetitions remained for the passive and active tasks for the healthy subjects, respectively.

We pre-processed all recorded EEG data offline using MATLAB (The MathWorks, Inc., USA), EEGLAB v14 toolbox (Delorme and Makeig, 2004) and the Fieldtrip toolbox (Oostenveld et al., 2011). As a first step, the EEG channel locations were aligned with the head model. Next, the EEG data were zero-phase band-pass filtered (0.5 to 40 Hz, FIR filter, order: 1691 and 87, respectively), cleaned from bad channels, and then re-referenced to the common average. On average, we removed 3.1 channels from the data. Next, the passive and active trials were divided into 1.25-second epochs and noisy epochs were visually identified and discarded from the data.

In the next step, we removed eye blinks and muscular artefacts using extended Infomax independent component analysis (ICA, (Bell and Sejnowski, 1995, Makeig et al., 1996)) on the combined passive and active EEG epochs. Artefact components were visually identified and removed based on from their power spectra and topographic activation. Finally, we used the *dipfit* function from the Fieldtrip toolbox to perform source localization by fitting equivalent dipoles to the remaining independent components in the individualized head models. The source space of the equivalent dipoles was restricted to nodes inside brain.

In the last step of selecting the functional motor target, we extracted the coordinates of the fitted dipoles based on: 1) the residual variance of the dipole had to be below 10 % (Delorme et al., 2012) and 2) differences in the alpha (8 to 12 Hz) and beta (14 to 30 Hz) power between passive and active trials, reflecting active motor engagement (Pfurtscheller et al., 1996). The middle grey matter node closest to the selected equivalent dipole location was used as the functional target. We converted the functional target’s coordinates to MNI (Montreal Neurological Institute) space coordinates and extracted the Brodmann area (BA) closest to the functional target to validate the source localization.

### 2.4 Simulation of tDCS

We also used SimNIBS 3.2 to extract the normal component of the electric field during conventional anodal tDCS in the anatomical and functional targets and to find optimal tDCS electrode configuration per subject. We used SimNIBS to optimize electrode configurations to maximize the normal component of the electric field, separately inside the anatomical and functional target. The normal component of the electric field was used as the outcome measure due to the hypothesized working mechanism of tDCS, which is polarity dependent (Jacobson et al., 2012, Radman et al., 2009, Rawji et al., 2018). We modelled all stimulation electrodes as rubber, circular disks (diameter: 10 mm; thickness: 3 mm; conductivity: 29.4 S/m). The simulated injected current was fixed at 2 mA.

#### 2.4.1 Conventional anodal tDCS electrode configurations

To assess the electric field strength in the anatomical and functional target with conventional anodal tDCS, we modelled an anode over the affected motor cortex at the C3 or C4 electrode location for the stroke subjects and always at C3 for the healthy subjects. The cathode was placed at the contralesional supra orbita (Fp1 or Fp2) for stroke subjects and at Fp2 for healthy subjects.

#### 2.4.2 Optimized tDCS electrode configurations

To find the maximum normal component of the electric field in each subject’s anatomical and functional target, we used SimNIBS to find the optimal electrode positions, without considering electric field focality. The full optimization procedure is described by Saturnino et al. (2019). Constraining focality would require additional assumptions on the allowable electric field strength in the brain areas outside the target. The stimulation electrodes were limited to 80 electrodes of the 10/10 system.

### 2.5 Statistical analysis

We compared the stimulation strength in the anatomical and functional targets by extracting the mean normal component of the simulated electric field within a 25 mm radius sphere centered around the grey matter node closest to the anatomical target and functional target coordinate, following (Mosayebi-Samani et al., 2021, Pimentel et al., 2013). We extracted the electric field from the anatomical and functional targets for conventional anodal tDCS and all optimized tDCS configurations.

We applied a linear mixed-effects model to assess the effect of optimized electrode configurations relative to conventional anodal tDCS. In this model, we set fixed effects for *stroke* (yes or no), *stimulation type* (conventional or optimized tDCS configuration) and *stimulation target* (anatomical or functional) and all two-way interaction terms between *stroke*, *stimulation type* and *stimulation target*. A random intercept was set for each subject per stimulation target to consider individual differences in stimulation strength. We performed posthoc tests to assess differences in electric field strength between stroke patients and healthy subjects for conventional anodal tDCS and optimized tDCS configurations for the anatomical and functional targets. The significance threshold was Bonferroni-corrected for multiple testing accordingly and set at 0.005.

## 3 Results

### 3.1 Participants

We analyzed data of twenty-one chronic hemiparetic stroke subjects (time post-stroke: 47 ± 35 months (mean ± standard deviation); age: 48 to 77 years (range); 6 females) and a mean FM-UE score of 44 (range: 8 to 66) and an average total EmNSA score of 33 (range: 9 to 40). Table 1 describes the demographics and functional assessments of all stroke subjects. Ten healthy age-matched subjects (51 to 75 years) underwent the same experimental protocol and served as a control group.

**Table 1:** Patient demographics and clinical assessment scores.

ID	Age (years)	Sex	Affected side	Timepost-stroke (months)	FM-UE	EmNSA
1	64	M	L	82	13	40
2	62	M	R	49	39	40
3	77	M	L	7	62	34
4	66	F	R	212	9	9
5	76	F	L	35	63	37
6	54	M	L	21	8	9
7	67	M	R	26	54	39
8	55	M	L	75	58	40
9	59	M	L	70	9	34
10	68	F	R	67	66	40
11	49	F	L	40	59	40
12	57	M	L	9	66	40
13	48	M	L	80	10	33
14	65	M	L	22	64	36
15	50	F	R	52	59	35
16	50	M	R	33	48	40
17	56	M	L	8	56	38
18	48	M	R	88	66	40
19	61	F	R	10	60	39
20	72	M	L	15	26	20
21	68	M	L	142	20	15

Sex (F: female, M: male); Affected side (L: left, R: right); FM-UE: Fugl-Meyer Assessment of upper extremity; EmNSA: Total score Erasmus MC Modifications to the Nottingham Sensory Assessment.

### 3.2 Stimulation target locations

We visually identified the anatomical target in the T1w MRI for 19/21 S subjects and all healthy subjects. No anatomical target could be identified in two stroke subjects because the lesion included the motor hand knob.

**Fig. 1:**
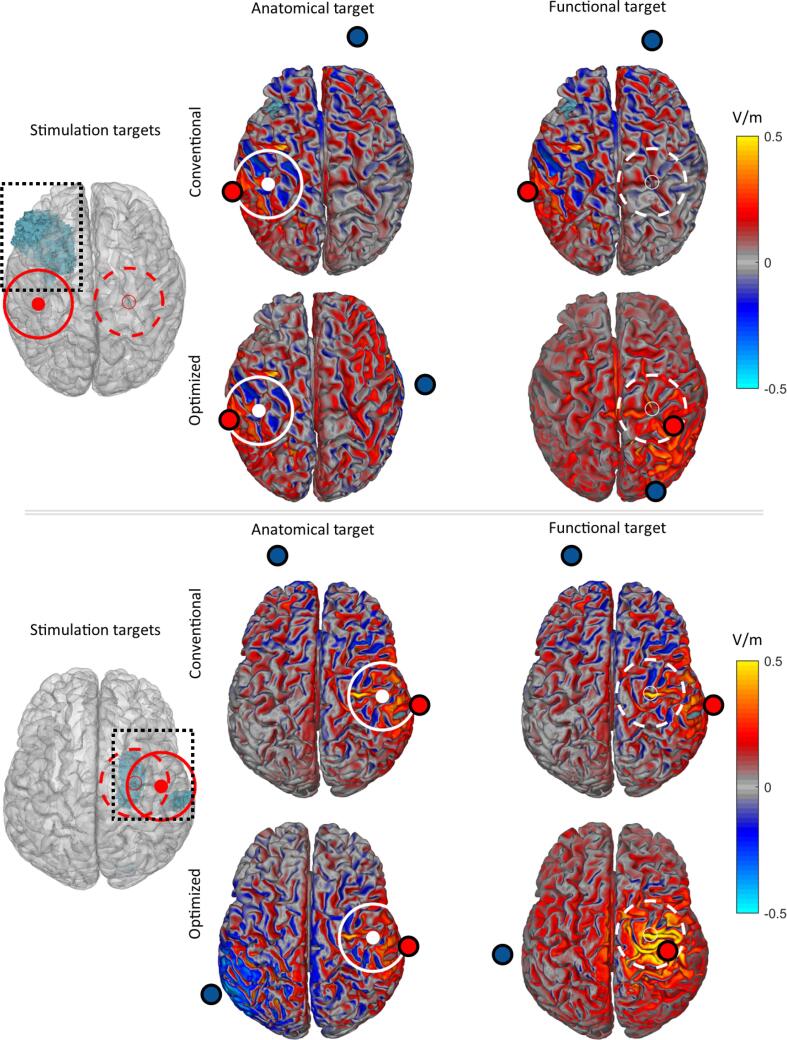
Brain model (left column) showing the stroke lesion in blue (boxed) and the consequent anatomical target (red circle), and the functional target (red dashed circle) of two stroke subjects. For the electric field during conventional tDCS electrode configuration, we show the normal component in the anatomical motor target (middle column) and the functional motor target (right column). For the electric field during optimized tDCS electrode configuration, we show the normal component of the electric field in the functional motor target and the anatomical motor target row 2 and 4); The white circles in each panel mark the intended stimulation target. (For interpretation of the references to colour in this figure legend, the reader is referred to the web version of this article.)

Due to excessive EMG artefacts, no functional target was identified from the EEG data in one stroke subject and two healthy subjects. In the healthy subjects, the functional target was always located in the contralateral hemisphere. In the stroke subjects, the functional targets were localized in different cortical areas of the ipsilesional and contralesional hemisphere (Fig. 1/Table 2): the premotor cortex/supplementary motor cortex (BA6), primary motor cortex (BA4), Wernicke’s area (BA22), intermediate frontal cortex (BA8), pars opercularis (BA44), primary somatosensory cortex (BA1), somatosensory association cortex (BA5), and the supramarginal gyrus (BA40). For 11/20 S patients, the functional target was located in the ipsilesional hemisphere, and in 9 patients in the contralesional hemisphere.

**Table 2:** Electric field normals within the anatomical and functional target during conventional stimulation and optimized tDCS.

			**Anatomical target**				**Functional target**			
			**Conventional**	**Optimized**			**Conventional**	**Optimized**		
**Lesion**^1^	**Func. target^2^**	**BA^3^**	**C3/C4-Fp2/Fp1**	**E [V/m]**	**A**	**C**	**C3/C4-Fp2/Fp1**	**E [V/m]**	**A**	**C**
R	R	4	0.062	0.064	C4	F9	0.030	0.051	CP2	T10
L	–	–	0.045	0.052	C1	T10	–	–	–	–
R	R	6	0.035	0.046	C4	*P*1	0.025	0.038	FC6	PO3
L	R	6	–	–	–	–	0.012	0.043	FC2	T8
R	L	6	0.055	0.061	C4	T9	0.013	0.045	C1	T9
R	R	6	0.041	0.060	*C*2	P9	0.038	0.056	*C*2	F10
L	R	6	0.071	0.076	C3	T8	0.005	0.052	*C*2	I2
R	R	6	0.057	0.066	C4	CP3	0.061	0.109	*C*2	TP9
R	R	22	0.068	0.074	*C*2	F10	0.026	0.044	CP6	T7
L	L	6	0.060	0.065	C3	TP8	0.013	0.044	Cz	P9
R	R	4	0.071	0.076	C4	TP7	0.064	0.069	C4	P9
R	R	8	0.077	0.083	C4	T7	0.061	0.095	FC4	I1
R	L	44	–	–	–	–	−0.013	0.062	FC3	C6
R	L	1	0.047	0.053	C4	TP7	0.006	0.048	C1	FT10
L	R	5	0.090	0.095	C3	T10	0.015	0.055	CP2	FT10
L	L	6	0.056	0.057	C3	F6	0.022	0.068	C1	P9
R	L	8	0.045	0.052	*C*2	FT9	0.002	0.060	FCz	T10
L	L	1	0.071	0.085	C1	FT10	0.017	0.035	C1	FT9
L	R	1	0.073	0.087	C1	PO10	−0.001	0.070	CP4	T7
R	L	1	0.049	0.052	C4	T9	0.010	0.060	C1	FT9
R	R	6	0.042	0.054	*C*2	T9	0.026	0.054	*C*2	T10
Healthy subjects										
–	L	8	0.069	0.074	C3	T10	−0.001	0.051	FCz	FT9
–	–	–	0.057	0.066	C1	T10	–	–	–	–
–	L	6	0.071	0.077	C3	P10	0.046	0.066	C1	I1
–	–	–	0.089	0.095	C3	P10	–	–	–	–
–	L	1	0.064	0.072	C3	FT9	0.060	0.074	CP3	T10
–	L	6	0.082	0.096	C1	P10	0.053	0.084	C1	PO9
–	L	6	0.093	0.100	C3	PO10	0.024	0.084	FC1	T9
–	L	6	0.093	0.094	C1	T10	0.083	0.087	C3	T10
–	L	40	0.057	0.061	CP1	FT9	0.062	0.067	CP3	FT10
–	L	4	0.086	0.089	C3	FT10	0.060	0.064	C3	T10

1 Impaired hemisphere (L: left; R: right: -: healthy subject); ^2^ Hemisphere containing the functional target; ^3^ Nearest Brodmann Area to the functional target; E: normal component of the electric field within the stimulated target; A: anode; C: cathode.

### 3.3 Electric field strength in stimulation targets

**Fig. 2:**
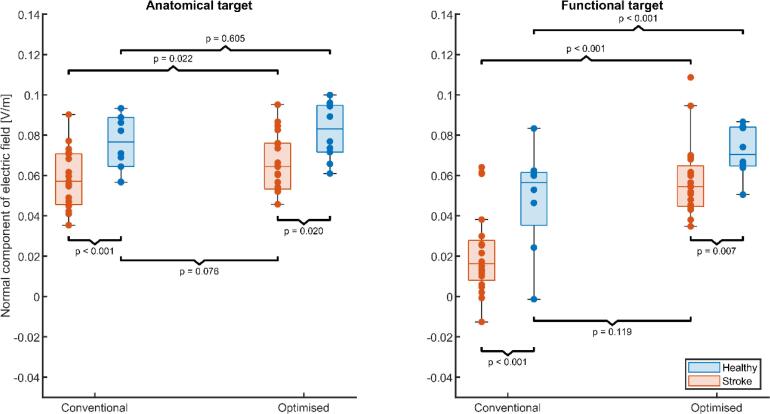
Stimulation strength in the anatomical target (left panel) and functional target (right panel) for stroke subjects (red) and healthy subjects (blue). Each box shows the median stimulation strength with the interquartile range; minimum and maximum data points (whiskers) and the outliers (data points beyond the maximum/minimum ± 1.5 times the interquartile range). Each panel shows the simulated normal component of the electric field within each target during conventional anodal tDCS and after optimization of the electrode configuration to maximize the electric field. Optimization based on individual characteristics increases the electric field in patients to similar levels as conventional anodal tDCS in healthy age-matched controls. (For interpretation of the references to colour in this figure legend, the reader is referred to the web version of this article.)

Fig. 2 shows the simulated normal component of the electric field in the anatomical and functional targets for conventional anodal tDCS and optimized tDCS, grouped by stroke and healthy subjects (see Fig. 1 for two exemplar stroke patients). In stroke patients, stimulation strength was highly variable for conventional anodal tDCS targeting the anatomical target, ranging from 0.035 V/m to 0.090 V/m. In the functional target of stroke patients, stimulation strength was distributed around 0.016 V/m, with stimulation intensities ranging from −0.013 V/m to 0.064 V/m. For 2 out of 20 S subjects, conventional anodal tDCS resulted unintentionally in negative stimulation of the functional target. Negative stimulation did not occur in the anatomical target for healthy subjects. However, negative stimulation occurred in the functional target (-0.001 V/m) in 1 of the 8 healthy subjects in which we identified a functional target.

In the anatomical target, posthoc tests on the linear mixed-effects model (see Table 3 for the estimated model coefficients) revealed a significantly lower mean stimulation strength (F_1,107_ = 12.18, p < 0.001) in stroke patients compared to healthy subjects. Optimization of the electrode configurations for the anatomical target marginally increased the stimulation strength for stroke patients (F_1,107_ = 5.42, p < 0.022) but not for healthy subjects (F_1,107_ = 0.27, ns). After optimization, the normal component of the electric field always had the correct, positive polarity. Furthermore, although optimization of the electrode configurations raised the electric field in the anatomical target of all stroke patients (mean ± sd: 0.062 ± 0.019 V/m), it remained lower than the electric fields acquired after optimization in healthy subjects (mean ± sd: 0.082 ± 0.014 V/m; F_1,107_ = 5.61, p = 0.020). However, the optimized electrode positions in stroke patients resulted in similar electric field strengths as non-optimized, conventional electrode positions (i.e. C3-Fp2) in healthy subjects (F_1,107_ = 3.21, p = 0.076).

**Table 3:** Summary of the estimates linear mixed-effect model that describes the mean normal component of the electric field in the anatomical and functional target for stroke patients and healthy subjects, shown in Fig. 2. This model served as the input for the post-hoc tests to determine whether the electric fields differed between stroke patients and healthy subjects for the different combinations of stimulation configurations and targets.

**Variable**	**Estimate [-]**	**SE**	**t(DF)**	**p**	**CI95**
Intercept	0.022	0.004	6.12 (107)	< 0.001	[0.015, 0.029]
Anatomical target	0.035	0.004	8.69 (107)	< 0.001	[0.027, 0.043]
Optimized	0.034	0.004	8.79 (107)	< 0.001	[0.027, 0.042]
Healthy	0.024	0.006	3.82 (107)	< 0.001	[0.011, 0.036]
Anatomical Target: Optimized	−0.025	0.005	−4.97 (107)	< 0.001	[-0.035, −0.015]
Anatomical Target: Healthy	−0.003	0.006	−0.52 (107)	0.601	[-0.014, 0.008]
Optimized: Healthy	−0.007	0.005	−1.24 (107)	0.219	[-0.018, 0.004]

In the functional target, posthoc tests on the linear mixed-effects model revealed that the electric field strength from conventional anodal tDCS was lower for stroke patients and healthy subjects (F_1,107_ = 14.59, p < 0.001). Optimization of the electrode configurations for the functional target increased the electric field strength for stroke patients (F_1,107_ = 77.30, p < 0.001) and for healthy subjects (F_1,107_ = 27.46, p < 0.001). After optimization, the electric field strength within the functional target was remained lower for the stroke patients (mean ± sd: 0.058 +- 0.018 V/m) compared to healthy subjects (mean ± sd: 0.013 +- 0.072 V/m; F_1,107_ = 7.52, p = 0.007). However, optimized electrode positions in stroke patients resulted in similar electric field strengths as non-individualized electrode positions in healthy subjects (F_1,107_ = 2.47, p = 0.119).

### 3.4 Optimal electrode configurations

The optimal electrode positions for both stimulation targets (anatomical and functional) and patient groups (healthy and stroke) are shown in Table 2 (for the resulting electric field of all optimal electrode configuration, see Supplementary Fig. 1). The variability in anode location was greatest within the functional target, with 12 unique locations for stroke subjects and 5 for healthy subjects. For stroke subjects within the anatomical target, the optimal anode was found at the C1/*C*2 electrode 7 times and 12 times for the conventional C3/C4 electrode. For healthy subjects, the C3 electrode was the optimal anode location 6/10 cases. For the optimal cathode locations, no clear pattern was found.

## 4 Discussion

This study investigated the variability in the electric field strengths generated in anatomical and functional targets by conventional tDCS configurations in stroke subjects and healthy age-matched controls. In addition, the study investigated the electrode configurations optimally stimulating these targets and the electric fields associated with these configurations. Our results show that the stimulation strength in the anatomical and functional target is lower for stroke patients than for healthy subjects when using conventional anodal tDCS. Optimizing the electrode configurations resulted in more different electrode locations in subjects with stroke than in healthy subjects, increasing the electric field strengths in stroke patients, although not to the same level as in healthy subjects, likely due to the stroke lesions. In healthy subjects, optimization of the electrode positions did not significantly increase electric field strengths in the anatomical target. Finally, optimized electrode configurations in the functional target resulted in higher and more consistent stimulation levels in both stroke patients and healthy age-matched subjects prevented negative stimulation, but electric field strengths in stroke patients remained lower than those in the healthy subjects. The above findings suggest that interindividual variability in electrical field strengths may have contributed to the lack of beneficial effects of tDCS found in clinical trials targeting the most-affected upper limb post stroke.

An important implication of our study is the need to individualize tDCS configurations in subjects with stroke, following from the difference in stimulation strength during conventional anodal tDCS between stroke patients and healthy subjects. The anatomical target (motor hand knob) is a frequently-used stimulation target in both healthy subjects and stroke patients. In healthy subjects, the simulated electric fields match previous modelling studies targeting the motor hand knob (Rawji et al., 2018) but stroke patients had lower electric field strengths overall. meta-analyses and reviews show that most clinical tDCS studies apply heterogenic stimulation paradigms, with C3 and C4 as anode locations as the only common factor between studies (Bornheim et al., 2022, Orrù et al., 2020). Our results argue that individualized electrode positions in stroke patients reduce the difference in electric field strength compared to healthy subjects while keeping stimulation current equal. While electric field strength in stimulation targets is only one factor that affects tDCS effects (López-Alonso et al., 2014) these findings indicate that individualizing electrode positions reduces one factor of variability, simplifying the comparison of reported tDCS effects in stroke patients and healthy subjects.

One factor commonly attributed to interindividual variability in stimulation strength is the local thickness of the CSF layer (Antonenko et al., 2021, Laakso et al., 2015, Mosayebi-Samani et al., 2021). Since the thickness of the CSF layer is age-dependent (Antonenko et al., 2021, Wanifuchi et al., 2002) and we compared the stroke patients with age-matched healthy controls, this might not be a key factor in our analysis. Additionally, optimized electrode positions resulted in lower electric field strengths for stroke patients as healthy subjects. Therefore, the achievable electric field strength for conventional anodal tDCS seems to be limited by lesion characteristics. Previous tDCS simulation studies have demonstrated that lesions can significantly alter local electric fields in the vicinity of a stimulation target, depending on the lesion size, conductivity and location relative to the stimulation target (Datta et al., 2011, Dmochowski et al., 2013, Johnstone et al., 2021, Minjoli et al., 2017, Piastra et al., 2021).

Considering the functional reorganization following stroke, the need to individualize the tDCS configurations becomes even more evident. Functional targets were found predominantly in or near parts of the sensorimotor network previously associated with functional reorganization following stroke (Jones and Adkins, 2015, Ward, 2004). As expected, conventional anodal tDCS – designed to target the ipsilesional motor hand knob – resulted in more variable stimulation strength and sometimes reversed polarity for the functional targets compared to the anatomical target. In particular, this was found in functional targets localized in the contralesional hemisphere. Optimization of electrode positions resolved both the variable magnitude and the polarity in both the anatomical and the functional targets in stroke patients and increased the electric field strength, but not to the same level as optimized electrode positions in healthy subjects. Solving unintended negative stimulation seems an important finding as negative stimulation of the functional target has potentially detrimental effects on neuroplasticity and motor learning. (Horvath et al., 2016, Stagg et al., 2011, Tazoe et al., 2014). While some patients may benefit from inhibiting the contralesional M1, others may benefit from stimulation of contralesional or ipsilesional motor regions (di Pino et al., 2014, Dodd et al., 2017). However, the most suitable stimulation locations and polarities should be determined per individual patient from functional neuroimaging to avoid promoting maladaptive reorganization of the motor system (Cunningham et al., 2015).

The findings of our study follow from several strengths that should be noted. First, our study combined structural and functional neuroimaging in a relatively large sample of 21 S subjects and performed a direct comparison with age-matched healthy subjects. Furthermore, we used 62-channel EEG to derive functional motor targets for tDCS, allowing us to investigate functional reorganization following stroke. Source localization and tDCS simulation were both performed in the same accurate, individualized finite element models.

Our study also has limitations. First, our source localization method did not result in functional targets for all subjects. As a source localization method, we fitted equivalent dipoles to independent components resulting from ICA. For some subjects, no motor task-related components were found due to excessive noise in the EEG recording. Furthermore, EEG source localization has a lower spatial resolution than alternative methods such as functional MRI or TMS-based identification of the stimulation target (Numssen et al., 2021). Therefore, it might be that the actual source of brain activity and modelled equivalent dipole did not completely match. The modelling inaccuracy is reflected by the MNI-transformed dipole locations, which were sometimes outside the sensorimotor network (i.e., BA8, 22, 44). However, visual inspection of these functional targets showed that these sources were close to sensorimotor regions. A previous modelling study showed that optimized electric field strengths are relatively robust to small variations of the target location (Dmochowski et al., 2013). Thus, we consider the source localization method a minor limitation for the interpretation of our results.

Our study included 21 patients with chronic stroke and 10 healthy age-matched but not gender-matched controls, which poses an additional limitation because structural MRI studies describe age-related and gender-related differences in the brain structure in the elderly (Greenberg et al., 2008). Furthermore, while the number of stroke patients is relatively high compared to similar modelling studies, the absolute number remains low. It is, therefore, unknown how our results generalize to larger samples. Patients with other lesion characteristics or different functional organization of the motor system likely require different stimulation configurations, emphasizing the need to individualize electrode locations to reduce intersubject variability in the electric field strength in the targeted brain region. An additional limitation follows from the small electrodes used in our simulations. The electrode size is of interest in tDCS research because smaller electrodes allow 1) more focal stimulation with higher electric fields than achievable with large sponge electrodes (Mikkonen et al., 2020) and 2) simultaneous recording of EEG during tDCS, an anticipated future combination with tES (Thut et al., 2017) However, more focal stimulation increases interindividual variability in electric field strength (Mikkonen et al., 2020), and it is thus unknown if our results apply to larger electrodes. Nonetheless, our results show less interindividual variability in the healthy control group than in the subjects with stroke, supporting our findings and modelling choice.

In our analysis, we focussed on the normal component of the electric field in each stimulation target due to the putative polarity-dependent effects of tDCS (Jacobson et al., 2012, Rawji et al., 2018, Stagg et al., 2011). However, MEP magnitudes in TMS studies were recently positively associated with particularly the magnitude and the tangent component of the electric field within the M1 (Weise et al., 2020). At this moment, it is unclear how such relationships fit within the polarity-dependent effects of tDCS. Exploring such relationships further requires combining simulation and optimization of tDCS with experimental paradigms, which was beyond the scope of the current study.

Finally, our individualized head models assumed known conductivities for all tissue types in the model. Literature shows that skull conductivity is highly variable (McCann et al., 2019) and negative correlation with electric field strengths (McCann and Beltrachini, 2021). Furthermore, we assumed CSF conductivity for the lesion, which might not represent all subjects in our sample, although commonly used (Datta et al., 2011, Minjoli et al., 2017). An individualized estimate of the skull and lesion conductivity are important next steps to improve the accuracy and validity of tDCS simulations (Piastra et al., 2021, van der Cruijsen et al., 2021).

In conclusion, our study shows that considering individual brain structure and functional motor targets is vital to applying tDCS in patients with chronic stroke and, to a lesser extent, also in healthy subjects. Without simulating tDCS in individualized head models, the electric field strength is lower and more variable in stroke patients, as may be the tDCS effects on clinical outcome measures at patient and group level. In future clinical studies, the effects of individualized tDCS targeting the motor hand knob and regions involved in functional reorganization remain to be tested.

## CRediT authorship contribution statement

**Joris van der Cruijsen:** Conceptualization, Formal analysis, Investigation, Methodology, Validation, Visualization, Writing – original draft. **Renée F. Dooren:** Conceptualization, Formal analysis, Investigation, Visualization, Writing – review & editing. **Alfred C. Schouten:** Supervision, Writing – review & editing. **Thom F. Oostendorp:** Supervision, Writing – review & editing. **Maarten A. Frens:** Supervision, Writing – review & editing. **Gerard M. Ribbers:** Supervision, Writing – review & editing. **Frans C.T. van der Helm:** Resources, Writing – review & editing. **Gert Kwakkel:** Resources, Writing – review & editing. **Ruud W. Selles:** Funding acquisition, Resources, Supervision, Writing – review & editing.

## Declaration of Competing Interest

The authors declare that they have no known competing financial interests or personal relationships that could have appeared to influence the work reported in this paper.

## Data Availability

The authors do not have permission to share data.
